# Towards a Procedure-Optimised Steerable Catheter for Deep-Seated Neurosurgery

**DOI:** 10.3390/biomedicines11072008

**Published:** 2023-07-17

**Authors:** Ayhan Aktas, Ali Anil Demircali, Riccardo Secoli, Burak Temelkuran, Ferdinando Rodriguez y Baena

**Affiliations:** 1 Mechatronics in Medicine Laboratory, Hamlyn Center, Imperial College London, London SW7 2AZ, UK; a.aktas20@imperial.ac.uk (A.A.); r.secoli@imperial.ac.uk (R.S.); 2 Department of Metabolism, Digestion and Reproduction, Imperial College London, London SW7 2AZ, UK; a.demircali@imperial.ac.uk (A.A.D.); b.temelkuran@imperial.ac.uk (B.T.)

**Keywords:** medical robotics, minimally invasive surgery, thermal drawing, needle steering

## Abstract

In recent years, steerable needles have attracted significant interest in relation to minimally invasive surgery (MIS). Specifically, the flexible, programmable bevel-tip needle (PBN) concept was successfully demonstrated in vivo in an evaluation of the feasibility of convection-enhanced delivery (CED) for chemotherapeutics within the ovine model with a 2.5 mm PBN prototype. However, further size reductions are necessary for other diagnostic and therapeutic procedures and drug delivery operations involving deep-seated tissue structures. Since PBNs have a complex cross-section geometry, standard production methods, such as extrusion, fail, as the outer diameter is reduced further. This paper presents our first attempt to demonstrate a new manufacturing method for PBNs that employs thermal drawing technology. Experimental characterisation tests were performed for the 2.5 mm PBN and the new 1.3 mm thermally drawn (TD) PBN prototype described here. The results show that thermal drawing presents a significant advantage in miniaturising complex needle structures. However, the steering behaviour was affected due to the choice of material in this first attempt, a limitation which will be addressed in future work.

## 1. Introduction

Minimally invasive surgery (MIS) has undergone significant growth in the last few decades due to its potential advantages for surgical outcomes, such as shorter recovery times and reduced hospitalization and tissue disruption [1,2,3,4]. Percutaneous interventions with needles are among the most common application areas of MIS [5]. Straight needles are frequently used in minimally invasive neurosurgery procedures, such as biopsies, blood sampling fluid delivery/extraction, and tumour ablation [6]. The effectiveness of these interventions depends on the surgical target being reached precisely and accurately without harming healthy tissues (i.e., with minimal damage to the surrounding tissue), which is frequently impaired by instrument design constraints. Since a straight needle may not be able to access a lesion on the first try, dynamic compensation for tip misplacement is impossible without retraction or reinsertion [7]; researchers have been developing robotic steerable needles with a range of designs [8]. When employing steerable catheters in soft tissue, it is important to consider the impact of tissue properties, such as inhomogeneity, anisotropy, and organ deformation, on their trajectory [9]. Active steering techniques are utilized to prevent misalignment due to factors such as operator error or catheter deflection [10]. Moreover, the development of steerable needles has the potential to expand the possibilities of minimally invasive procedures by enabling access to previously challenging targets with complex anatomical features [11]. In the literature, we found various needle-steering technologies available that enable navigation through tissues along curved paths [12,13]. These technologies are needle-steering control via concentric tubes [14], needle steering via the lateral motion of the needle [15], fixed-shaped bevel-tip needle steering [16], pre-curved stylets [17], tendon actuation-based needles [18], shape memory-actuated needles [19], and magnetically driven flexible needles [20]. One particular design, the programmable bevel-tip needle (PBN), has recently been deployed in vivo for the first time in order to evaluate the feasibility of convection-enhanced delivery (CED) of chemotherapeutics through preferred curvilinear paths that align to specific anisotropic brain structures [5].

**Figure 1:**
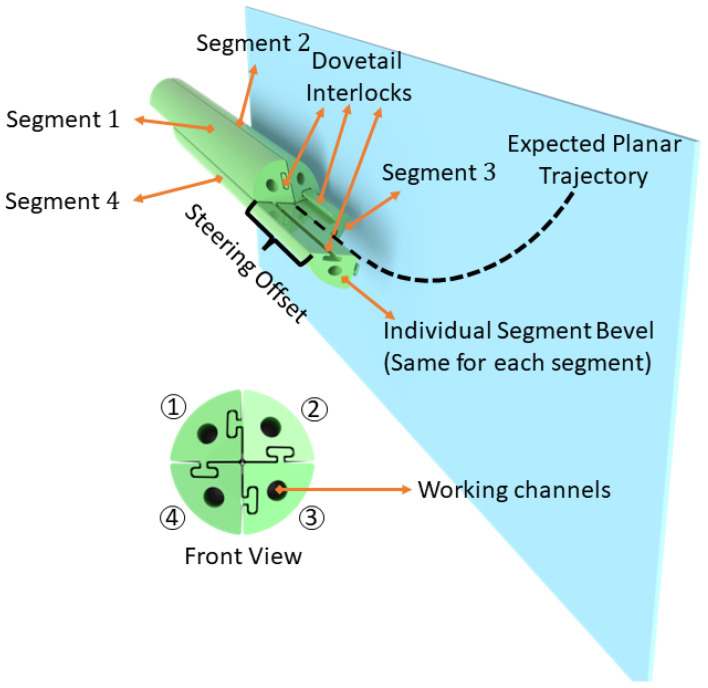
The programmable bevel-tip needle (PBN) steering concept applied to a four-segment probe.

The bio-inspired PBN design features multiple bevel-tip segments (generally four segments with multiple lumens per segment) held together through an interlocking mechanism. It uses the relative motion of the segments to generate a dynamic offset at the tip that enables steering during the insertion process, as illustrated in Figure 1. Control over the shape of the tip is achieved by adjusting the relative insertion of the four segments, thereby influencing the needle’s steering behaviour within the tissue. The interlocked segments can slide relative to one another, and the segment in the most forward position is the “leading segment” for the movement (Segment 4 in Figure 1). The steering offset affects how much the segment deflects due to tissue interaction forces during insertion. The radius of curvature during probe insertion depends on this offset. By creating an offset between the segments and pushing forward, a desired trajectory can be obtained. The offset parameter affects the radius of curvature when inserting the probe [21].

Starting a decade ago, the first generation of PBN prototypes had a 12 mm diameter and were manufactured by rapid prototyping technology using rubber-like materials, such as TangoBlack and VeroWhite [22]. The manufacturing of such complex designs finally reached a clinically viable size of 2.5 mm in diameter through a conventional extrusion manufacturing process [23], the gold standard for medical catheter production. The PBN segments were produced in a coloured bio-compatible polymer, each with two working channels. This version was used as an implantable device during an in vivo study on the ovine model, demonstrating the clinical viability of the design for the first time [5].

Though clinically applicable, a further size reduction would be necessary for applications involving deep-seated tissue structures, such as drug delivery and laser interstitial thermal therapy (LiTT) [24]. Due to the complex design of PBNs, standard manufacturing methods have limited capacity to achieve further size reduction. Therefore, alternative solutions to decrease the overall size need to be explored. In this study, we introduce a new manufacturing method for PBNs that employs thermal drawing technology [25] and demonstrates the potential to manufacture small-sized, complex catheter cross-sectional geometries with unprecedented detail.

Thermal drawing is a manufacturing process that produces longitudinally homogenous fibres while keeping the cross-sectional integrity of the preform. It achieves this by heating a glass or polymer preform to a glassy state and applying tension to draw it into long, thin fibres [26]. The preform is typically inserted into a furnace heated to high temperatures. When it has softened (glassy state), draw tension is utilised to manage the pulling speed, which causes the diameter to decrease and elongation to occur. Using the thermal drawing method, it is possible to accurately manipulate dimensions and create fibres with specific outer and inner diameters and wall thicknesses [27]. Three-dimensional (3D) printing technology enabled the creation of a preform with a complex cross-section, which could have been more expensive and challenging to fabricate using other approaches, such as moulding [28]. Recent advancements in 3D printing technology allowed the creation of another preform with a complex cross-section [29], potentially expanding the applications of thermal drawing to catheter production. This approach offers opportunities for design flexibility and material selection, leading to potential advancements in catheter manufacturing.

Thermally drawn, multi-material fibres with varying geometries, material compositions, and functionalities have been achieved in the past 20 years. This method was previously employed at the micro/nano-scale to produce complicated and asymmetrical structures at large scales. The first type of multi-material fibres demonstrating large photonic band gaps date back to 2002 [30,31]. Fibre technology involves many different technologies, such as photodetection, thermal sensing, chemical sensing, optical communication, and microfluidics [32,33,34,35]. In addition, thermal drawing can be used for electronics (e.g., fabric-based and wearable energy-storing systems) and functional textiles [36]. With the advent of additive manufacturing techniques, it became possible to fabricate complex and arbitrary structures at the micrometre scale. Due to these advantages, the thermal drawing technique was employed here to produce each PBN segment, as it allowed miniaturization of the complex cross-section beyond what has been possible until now with conventional manufacturing processes. We used a 3D printer (i.e., fused filament fabrication) to create complex geometries with cavities and produced increasingly small prototypes through an iterative refinement process.

This paper describes the fabrication of a sub-millimetre-size needle segment by thermally drawing a 3D printed preform (further reducing the needle size by approximately 50%) and presents comparative characterisation tests against the latest PBN described in [23]. These characterisation tests were performed as described in [37,38], with refinements to the process that included flexural stiffness and tensile strength assessments. A stereo camera pair was used for estimation of the curvature of the needle to investigate the relationship between segment offset and curvature.

This paper is organised as follows. Section 2 provides detailed information about the current needles and the characterisation methods used for mechanical testing and bending performance assessment. The experiments conducted are explained in Section 3. In Section 4, the experimental results are summarised. Finally, this paper concludes with a discussion of the advantages and disadvantages of each prototype and manufacturing method, with clear implications for the future of these technologies.

## 2. Materials and Methods

### 2.1. Catheter Design and Manufacturing

#### 2.1.1. Extrusion-Manufactured (EM) PBN Catheter

**Figure 2:**
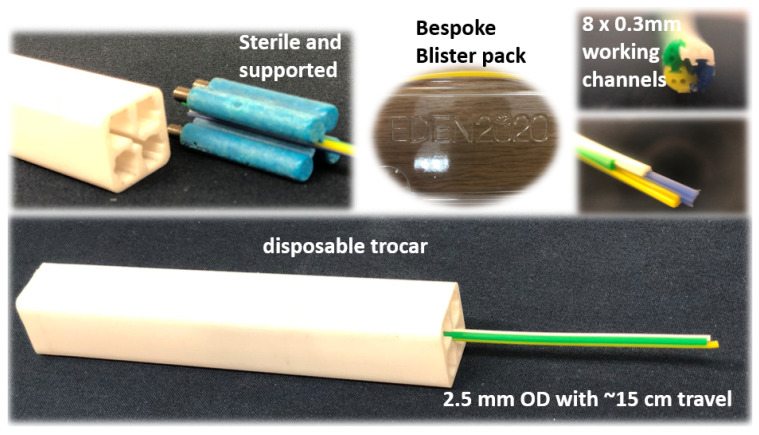
Extrusion-manufactured (EM) biocompatible PBN catheter. From top left clockwise: wing mechanism, bespoke blister pack, front view, and the catheter in the trocar.

The EDEN2020 modular robotic environment for precision neurosurgery used for the PBN prototype here is comprehensively described in [5]. The clinical experiments used a four-segment PBN catheter with a 2.5 mm outer diameter, as shown in Figure 2. The catheter was manufactured using extrusion because of the advantages of its capacity to create various cross-sectional forms and shapes. The extrusion of the PBN segments [39] was started by feeding the polymer granules into the extruder head. Inside the extruder, the polymer melted due to the combined heat generated by the heating element and the shearing energy produced by the granule and screw. To create the interlocking mechanism, the dove-tail, and the cross-sectional pattern for the PBN segment, a specially designed die was used to shape the extruded polymer. A mandrel was utilised to create the working channel within the die. Xograph Healthcare Ltd. fabricates needle segments via extrusion in medical-grade poly(vinyl chloride), which has Shore 89 A hardness. Colour particles were added to the mix in order to achieve a colour-coded design, with each segment easily identifiable in the operating theatre. The manufactured PBN segments were nano-coated with poly(para-xylylene) to ensure biocompatibility and minimal friction between the segments while the segments are sliding with respect to one another. Figure 2 shows the bespoke catheter segments and their cross-sectional view. In order to minimise the chance of buckling outside of the gelatine, a trocar with a 2.7 mm inner diameter was used. Each catheter segment contains a male and a female part that interlock with adjacent segments. Each segment has two 0.3 mm diameter working channels for sensorisation and instrument delivery, and the segment tips are bevelled at a 45${}^{\circ }$ angle from the neutral axis to aid the insertion process, as described in other studies (e.g., [23,39]).

#### 2.1.2. Thermally Drawn Catheter (TD)

Due to its unequivocal advantages when working at tiny scales, we thermally drew the 3D printed preforms to manufacture each PBN section with a miniaturised PBN design. Figure 2 shows the cross-section of the EM PBN catheter, which has quadrant-shaped segments with dove-tail interlocking mechanisms on the sides and two working channels. To achieve smaller dimensions with a functional channel, various design strategies were explored in the preform-creation process, including reducing the number of working channels from two to one. Furthermore, preform design was iteratively optimised to achieve the intended segment dimensions and the sliding performance for the interlocking mechanism by considering the influence of thermal expansion on prototype behaviour. Standard computer-aided design (CAD) software (SolidWorks, Dassault Systemes, France) was used to construct the 3D printed preform design. Several design strategies were considered during preform creation and iteratively optimised to achieve the intended segment dimensions and the sliding performance for the interlocking mechanism by taking into account the influence of thermal expansion.

A commercially available Ultimaker 3 Extended printer (UltiMaker BV, Geldermalsen, The Netherlands) was used with poly carbonate (PC) material (Ultimaker PC Transparent, 2.85 mm filament), which has Shore 82 D hardness and a 0.4 AA print core (non-abrasive plastics). The layer thickness was chosen as 0.1 mm with 100% infill density.

**Figure 3:**
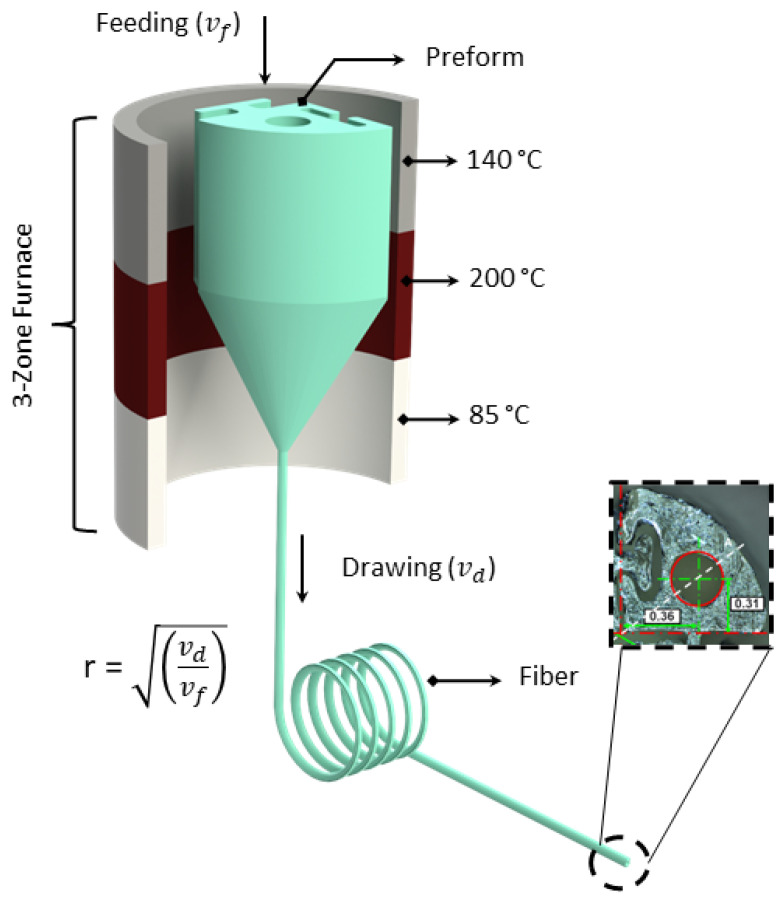
Thermal drawing process with 3D printed preform; on the right side, the cross-section of the fibre can be seen.

In contrast to the default speed of 250 mm/s, the print core travel speed was adjusted to 40 mm/s to create smoother surfaces. For transparent PC printing, the following temperatures were taken into account: nozzle temperature $T_{n}=270$ °C and bed temperature $T_{b}=107$ °C. The dimensions of the 3D printed preform were 40 mm for the diameter and 100 mm for the length. The central lumen of the preform was 8.6 mm in diameter, and the targeted draw ratio was 40 to reach 0.21 mm central lumen. Feeding the preform into a three-zone furnace with a speed of $v_{f}=2.5$ m/min and pulling the fibres at $v_{d}=1.4$ m/min, the 4 cm diameter preform with a 10 cm drawable preform length yielded 160 m of PBN sections. The temperature of the three-zone furnace used to thermally draw the fibre was as follows: top zone—140 °C, middle zone—200 °C, and bottom zone—85 °C. A schematic representation of the thermal drawing process can be seen in Figure 3.

**Figure 4:**
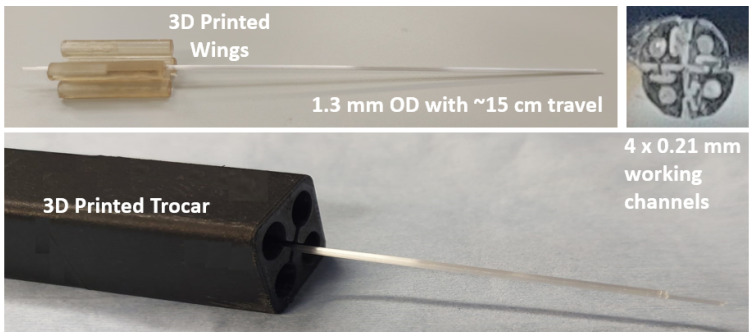
Thermally drawn (TD) programmable bevel-tip catheter. From top left clockwise: wing mechanism, front view, and the catheter in the trocar.

The thermal drawing process produced a long PBN-shaped fibre segment with a radius of 0.65 mm and, afterwards, the long fibre segment was trimmed to the desired length of 200 mm, which was chosen to match the existing prototype described in [39]. As in many previous studies, each short segment was fixed to 3D printed "wings", where were used to assemble each with an actuation mechanism, and needle assembly was completed manually by interlocking four individual segments. Figure 4 shows the TD segments and their cross-sectional view. In order to minimise the chance of buckling outside of the gelatine phantom, a specially designed trocar with a 1.3 mm inner diameter was constructed and employed in the characterisation experiments. Each catheter segment had a 1 × 0.21 mm diameter working channel for sensorisation and clinical applications, and the segment tips were bevelled at an angle of 45° from the neutral axis.

### 2.2. Characterization Methods

#### 2.2.1. Mechanical Feature Testing

Structural tests were performed to measure the material characteristics of the two PBN designs used in our in vivo work and the thermally drawn prototype described here. A flexural rigidity test was used to measure the needle sensitivity to medium changes. This sensitivity can either complement the needle steering or act as a competing mechanism that works against it depending on the steering strategy used for the needle [8]. Consequently, three-point bending tests were conducted to measure the flexural stiffness of the EM PBN and thermally drawn segments with a 30 mm specimen length. The test was performed with a 10 N Instron load cell (INSTRON, Norwood, MA, USA) with a cross-head velocity of 1 mm/min.

**Figure 5:**
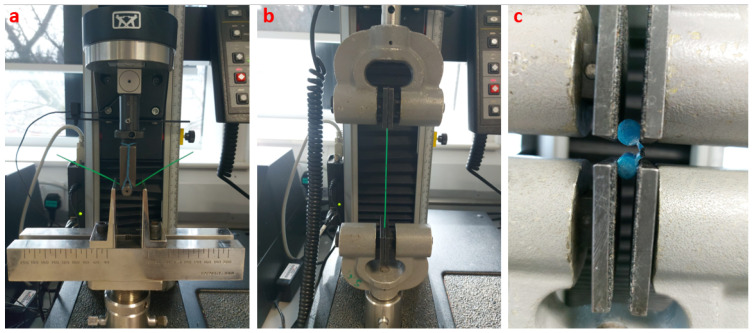
(**a**) Flextural stiffness test, (**b**) tensile strength test, (**c**) interlocking breakout force test.

In addition, tensile tests were conducted to determine the tensile stress–strain behaviour of each PBN. The tests were performed using the same samples with a 1 kN Instron load cell with a 50 mm/min pulling velocity. Lastly, the holding capacity of the interlocking mechanism was investigated, as unwanted separation of the segments would lead to the failure of the PBN. A tensile test was employed to measure the strength of the interlocking mechanism for each prototype. The test was performed on two interlocked segments fixed together through a holder attached to the wings. The two segments were subsequently pulled out from one another with a 1 mm/min velocity. The force at the disconnection was identified by a sudden decrease in the tensile force and was taken as the breakout force. Each experiment was repeated three times. The test setups are shown in Figure 5.

#### 2.2.2. Curvature Estimation Method

**Figure 6:**
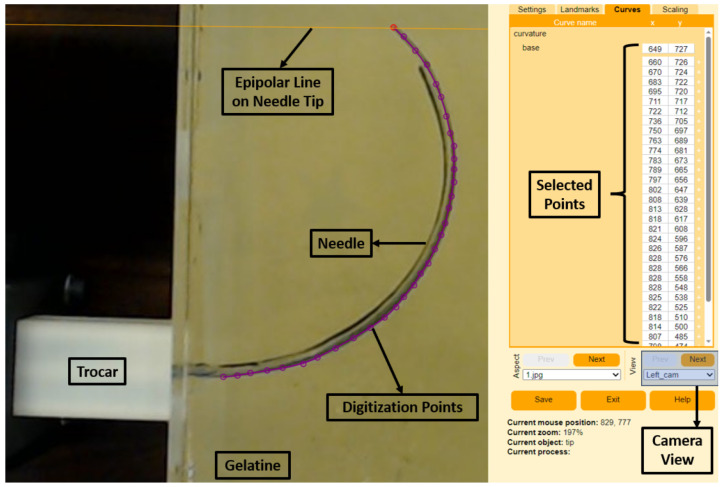
StereoMorph digitizing application with the right camera view of the EM PBN. The selected points on the picture are on the left-hand side, and corresponding selected digitization points are on the right-hand side.

The 3D curvatures of each needle were collected using a stereo camera setup following camera calibration, shape digitization, and shape reconstruction using the R package StereoMorph [40]. The stereo camera setup consisted of two fixed C920 HD Pro cameras (Logitech Inc., Lausanne, Switzerland) with fixed focal lengths and overlapping fields of view, calibrated using a classic checkerboard pattern. The calibration of the two stereo cameras was undertaken using StereoMorph’s calibration process as described in [40]. Following each insertion, the needle was photographed using the stereo cameras, and the needle shape was manually digitized in each camera view using the StereoMorph digitizing application. We used 32 evenly spaced points to capture the curvature of each shape. Figure 6 shows an example of the digitizing tool used for one of the experiments. The purple dots show the selected digitization points from the right-side camera, and selected points appear on the right-hand side of the program. In addition, the application shows the epipolar lines from the base and tip of the needle to match the left and right camera images. Following point selection, the digitization points were interpreted using the Bezier curve approach, which is embedded in the application. The digitized curvatures were then reconstructed from base to tip in 3D according to the calibration coefficients identified offline. Finally, refractive index correction was taken into account to compensate for the measurements obtained from the gelatine medium.

## 3. Experimental Validation

**Figure 7:**
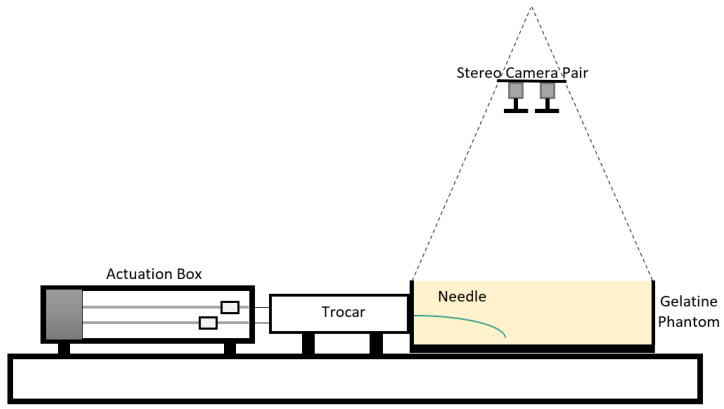
Diagram of the experimental setup: the actuation box moves the needle in the trocar, the needle is inserted into the gelatine phantom, and the stereo camera set is placed on the top of the gelatine box.

**Figure 8:**
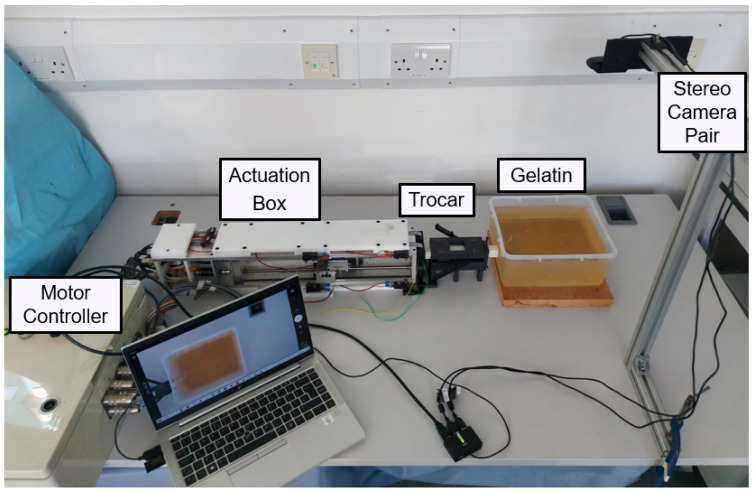
Annotated diagram of experimental setup.

The steering characteristics of both PBNs were assessed with a series of experimental needle insertions. These were performed using the same technique as in earlier investigations [22,38,41]. Figure 7 shows a diagram of the needle-steering setup for the characterisation tests. The insertions were performed using four linear actuators, controlled via software developed in house [42]. Each linear actuator was connected to a needle segment’s wing via a nitinol rod (which allowed sliding between the segments) travelling at a fixed speed of 1 mm/s, as in previous studies [22,37,38]. Each segment was enclosed within a 3D printed trocar core to preserve the needle segments’ alignment before insertion. The experimental setup is shown in Figure 8. The maximum achievable curvature for a range of offsets was used to compare the needles’ steering capabilities, including the minimum achievable radius of curvature [38]. The characterisation tests were conducted in a temperature-controlled environment at 20–21 °C using a 6% by weight bovine gelatine phantom (Chef William Powdered gelatine) [37], which is an acceptable first-order approximation of human brain white matter [43]. All needle segments were initially aligned and inserted 20 mm into the phantom. The segment or segments extending furthest were kept fixed, and the remaining segments were driven back to achieve specified offset configurations. Then, all four linear actuators were synchronously driven to achieve insertion of 110 mm, as in previous characterisation work [38].

Insertions were performed with configurations with a single segment positioned forward and two segments positioned forward to evaluate the achievable curvature performance in all four planes, with increasing insertion offsets of 5, 10, 15 and 20 mm. A minimum of 10 insertions were carried out for each tip configuration. The achieved needle trajectories were measured using the calibrated stereo camera pair after each insertion.

Calibration of the stereo cameras was undertaken with an 8 × 11 checkerboard and StereoMorph’s digitising app, resulting in a 0.423-pixel epipolar mean error. At the end of each insertion, we manually identified the curvature projections for the stereo camera images to be reconstructed in 3D using the StereoMorph digitising tool, as in Figure 6. StereoMorph reconstruction mode was used after point selection to obtain needle curvatures in 3D. In order to account for the refraction at the air–gelatine interface, a universal algorithm [44] was used because the stereo camera calibration was performed in the free air medium, whereas the experimental trials were conducted in a gelatine medium.

Following refraction correction, the curvature vector $\kappa_{exp}$ was estimated from the resulting trajectories. Murthy’s 3D circle fit function [45] was used to estimate the best-fit radius (*R*) of each curvature, from which the magnitude of the curvature could be computed as κ=1/R. The steering and horizontal planes (where the needle was originally placed) were used to calculate the insertion angle (β). The resulting curvature vector for each insertion was calculated as in [38].

$$\kappa_{exp}=\kappa \left[\begin{array}{c} \cos\;\left(\beta \right)\\ \sin\;\left(\beta \right). \end{array}\right]$$

## 4. Results

### 4.1. Mechanical Features

**Figure 9:**
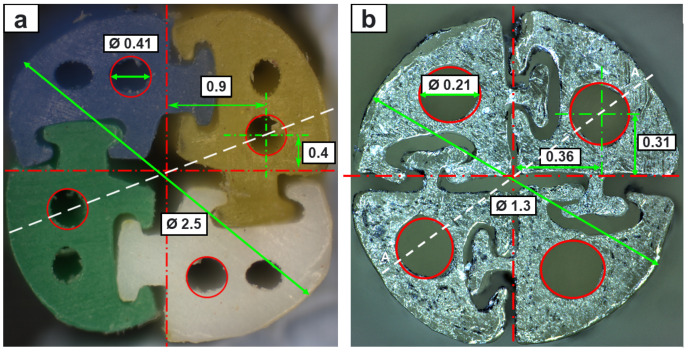
(**a**) Cross-section of EM needle with its dimensions, (**b**) cross-section of TD needle with its dimensions.

Figure 9 shows a cross-section comparison of the extrusion-manufactured (EM) and TD PBN segments viewed under a microscope. The findings indicate that this method reduced the diameter to about half of the state-of-the-art size while retaining full functionality and four working channels. We opted to halve the number of lumens per segment in order to maintain a 200-micron working channel in the smaller PBN for eventual integration with our fibre brag grating-based experimental setup [42].

The mean flexural stiffness, tensile stress, and interlocking mechanism breakout force values for the EM and TD PBN segments (2.5 mm and 1.3 mm diameters) are given in Table 1. Segment rigidity strongly influences the curvature performance during the needle insertion stages. The thermally drawn material had higher stiffness than the plastic employed with the extrusion method. As a result, the catheter curvature performance worsened despite the smaller diameter, while the interlocking strength was improved. Specifically, with the 1.3 mm prototype, the steering performance was 30% worse, while the interlocking strength was 50% better. Qualitatively, the thermally drawn segments worked more efficiently, moving with respect to one another more fluidly and predictably.

### 4.2. Offset vs. Curvature

**Figure 10:**
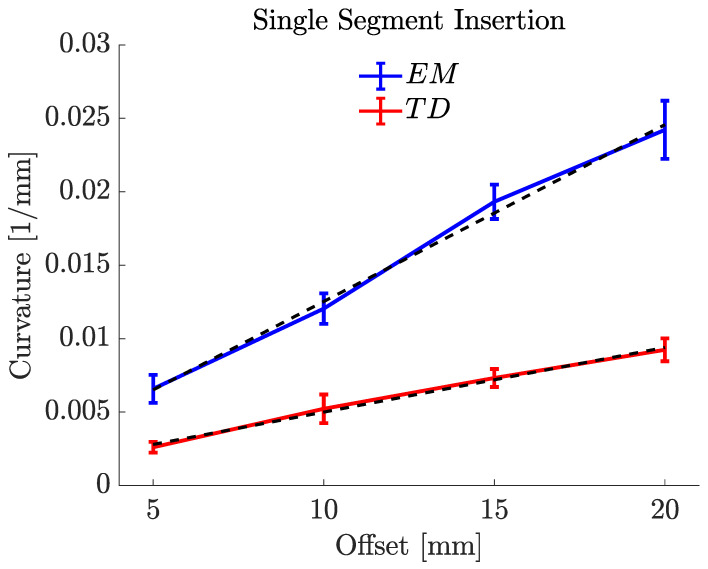
Single-forward-segment insertion characterization results for 5% gelatine sample (offset–curvature).

**Figure 11:**
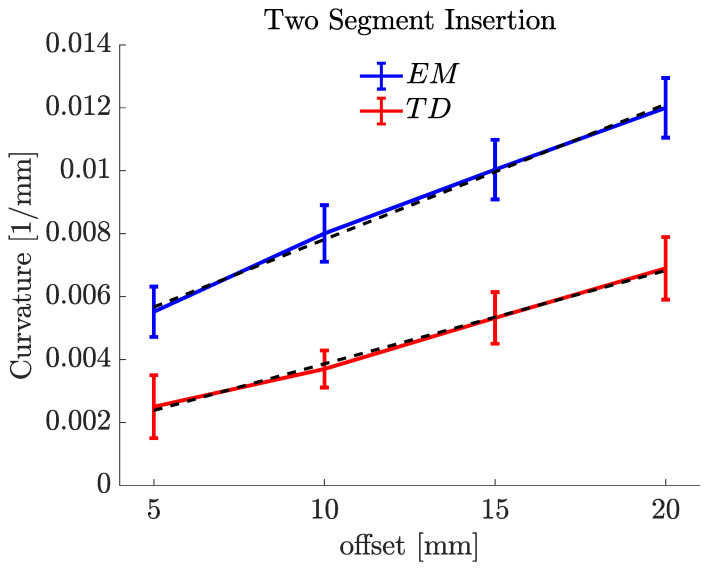
Two-forward-segment insertion characterization results for 5% gelatine sample (offset–curvature).

To assess the steering behaviour of the new prototype against our state-of-the-art preclinical PBN, two experiments were performed using the EM and TD PBNs: insertions with a "single leading segment" and "two leading segments". Figure 10 and Figure 11 display the mean values and standard deviations for 10 experiments for each test. For each, a linear fit of the curvature data in relation to the steering offset is also depicted.

Figure 10 displays the offset–curvature characteristics for a single-segment insertion for both needles. The minor disparity between the curvature values for the positive and negative offsets may have been caused by needle torsion, positioning uncertainty during the insertion process, and deformation. The maximum curvature achieved for the EM PBN was $0.0242\pm 0.003\;{\mathrm{mm}}^{-1}$, which corresponded to a radius of curvature of 41.307mm. The resulting curvature of this needle was substantially higher than the value of $0.0192\;{\mathrm{mm}}^{-1}$ previously reported in [38] under similar settings. This result was expected due to the use of a less stiff material resulting from the addition of colour pigments and the addition of a working channel per segment (the PBN in [38] only possessed one working channel per segment). The maximum curvature achieved for the TD PBN was $0.0092\pm 0.001\;{\mathrm{mm}}^{-1}$, which corresponded to a radius of curvature of 109.113mm. Table 2 shows the experimental results for single-segment insertion. The first column shows the offsets for each needle, and the last three columns of Table 2 report the mean curvature (*κ* (mm^−1^)), the radius of curvature (*R* (mm)), and angle of insertion (β).

The same experiments were conducted with two forward segments, as shown in Figure 11. The maximum curvature achieved for the EM PBN with two forward segments was $0.0121\pm 0.001\;{\mathrm{mm}}^{-1}$, which corresponded to a radius of curvature of 82.644mm. The maximum curvature achieved for the TD PBN with two forward segments was $0.0069\pm 0.001\;{\mathrm{mm}}^{-1}$, which corresponded to a radius of curvature of 144.921mm. Table 3 shows the experimental results for the two-segment insertions. The first column shows the offsets for each catheter, and the last two columns of Table 3 report the mean curvature (κ (mm)) and radius of curvature (*R* (mm)). The insertion angle is not reported because the curvatures here were measured in 2D, as they were performed in the plane normal to the camera line of sight. The reason for the difference between the single segment and the two segments is discussed in our previous study [37]. It relates to the effective stiffness of the programmable bevel achieved with two segments compared to just one. In Figure 10 and Figure 11, there is a linear relationship between curvature and offset, and we can see this with both needle types.

## 5. Discussion

**Figure 12:**
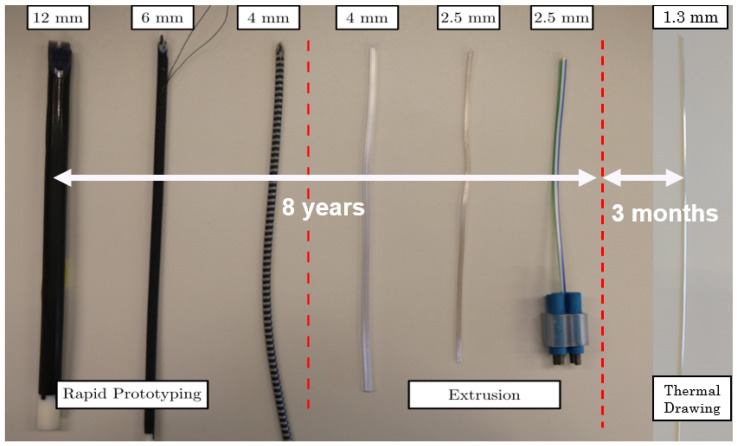
Evolution of PBN prototype sizes over time [39].

This study presents an alternative technique to manufacture complex-shaped needles and catheters with sizes exceeding conventional extrusion limits. Using thermal drawing, it was shown that it is possible to produce sub-millimetre segments with high tolerance and precise shape control. Considering the length of time needed to miniaturise each successive PBN prototype (Figure 12) using conventional manufacturing techniques, it took over eight years to reduce the size of our very first 12 mm prototype down to 2.5 mm. However, the thermal drawing method allowed us to achieve a 50% reduction over three months, an achievement that will now enable the application of the steerable system described in [5] to a broader range of diagnostic and therapeutic interventions.

Inaccuracies in the interlocking mechanism exist, but they do not affect the sliding behaviour or breakout strength between the segments, confirming the potential of thermal drawing as a substitute for extrusion. Additionally, when comparing the thermal drawing method to extrusion, the former does not need additional post-production processing (e.g., nanocoating to improve the sliding behaviour and to make the segments biocompatible), representing a marked advantage. Moreover, the interlocking strength of the TD catheter is 50% stronger, meaning segment separation will not be a problem as we reduce the outer diameter further, and the sliding behaviour of the TD segments is markedly better.

On the other hand, curvature characterization results showed that the new TD PBN performance is worse than that of EM PBNs despite almost halving the needle diameter. This demonstrates the importance of material stiffness for curvature performance. The TD PBN steering performance is low because the material (poly carbonate) used for thermal drawing has higher stiffness compared to EM PBN material poly(vinyl chloride). Poly carbonate (PC) was chosen for this study due to its availability and local experience with the manufacturing technique. PC is an ideal material for medical devices due to its superior mechanical strength, thermal stability, and biocompatibility. Previous experiments have shown that PC also provides reliable sliding performance, which is essential for catheters to navigate smoothly through blood vessels and other anatomical structures. However, the inherent specifications of PC bring about limitations for the steering behaviour of the catheter. Since PC is a rigid thermoplastic, it is less flexible when compared to other materials used for catheter manufacturing, such as polyurethane. Refining the stiffness profile with softer materials and tuning the thermal drawing process will be a focus of future work, enabling us to match and exceed steering performance.

However, exploring alternative materials for catheter manufacturing presents some particular challenges that must be taken into account. One of the key challenges is finding materials that are compatible with the thermal drawing process. The material choice for the thermal drawing process is limited mostly to amorphous thermoplastics. In addition, the selection of a catheter’s material is influenced by factors like bio-compatibility, mechanical attributes, flexibility, and sterilisation compatibility, making the process even more taxing.

Moreover, modifying and optimizing the manufacturing process to use alternative materials involves adjusting the thermal drawing parameters. This includes modifying the heating profiles, drawing speeds, and cooling methods to ensure the catheter meets the desired characteristics and dimensions. Although material exploration has its challenges, the potential rewards in terms of better catheter performance and patient outcomes make it a direction worth taking in the search for novel catheter designs.

The advantages of the thermal drawing method can be summarized as:

- Improved sliding behaviour: Enhances the sliding behaviour of the catheter segments, reducing the risk of tissue damage during insertion and removal;
- Stronger segment interlocking: Creates stronger interlocking segments, minimizing the likelihood of segment separation;
- Smaller catheter size: Enables the production of small-size catheters, beneficial for MIS procedures and patient comfort;
- Design flexibility: Offers adaptability in catheter designs to meet various surgical needs;
- Reduced post-production processes: Eliminates the need for additional post-production processes to improve sliding behaviour and ensure biocompatibility;
- Cost-effectiveness: Reduces manufacturing costs by eliminating complex tooling and moulds used in conventional techniques.

The disadvantages of the thermal drawing method can be summarized as:

- Lower steering performance: The material’s stiffness in thermal drawing can limit the catheter’s manoeuvrability when navigating complex anatomical structures;
- Limited material selection: The method is primarily suitable for amorphous thermoplastics, limiting the choice of materials for catheter manufacturing;
- Challenges in material characterization: Extensive testing and characterization are necessary to evaluate material properties for thermal drawing;
- Process optimization: Achieving consistent and reliable size and catheter features requires meticulous parameter adjustments (though this is true for other manufacturing methods too);
- The complexity of preform fabrication: achieving preforms with desired properties through 3D printing and catheter design expertise can be challenging.

The TD needle should also be less disruptive during the insertion process, as the smaller outer diameter will impact the size of the track left behind. Visual inspection showed that the TD needle significantly reduced damage at the entrance and along the needle track. However, in future work, quantitative analysis of the same will be carried out, as in Leibenger’s study [46], to confirm this conclusively.

## 6. Conclusions and Future Work

This paper describes ongoing research on using the thermal drawing method to produce complex-shaped catheters at the sub-millimetre scale with a specific application in needle steering. Specifically, it demonstrates the method’s application to needle steering through the design, manufacture, and characterisation of a 1.3 mm programmable bevel-tip needle able to meet the size requirements for deep-seated neurosurgical interventions. Through mechanical tests, we conducted a comparative analysis of the new prototype and our state-of-the-art preclinical system manufactured commercially via conventional extrusion. Moreover, fixed-offset experimental insertions into a phantom were performed to compare and contrast the steering capabilities of the new PBN. The main results of our experiments showed a significant achievement in size reduction, reducing the outer diameter by 50 per cent (from 2.5 mm to 1.3 mm). However, it was determined that the material employed during the thermal drawing process caused shallower steering behaviour. This finding highlights the importance of conducting a thorough investigation to determine how material choice affects a needle’s curvature performance. In our future research, we will conduct a thorough investigation of various material options for the thermal drawing process in order to address the shallower steering behaviour seen in the current prototype. We seek to improve the curvature performance of the needle and produce bends that are more precisely executed by carefully choosing the appropriate materials.

Furthermore, we plan to improve the catheter manufacturing process by investigating different preform moulding materials and techniques. This will help to make the manufacturing process more efficient and enhance the catheter’s steering performance.

The size reduction achieved and current research projects show significant potential for improving the effectiveness of neurosurgical procedures. Our research aims to go beyond material selection and manufacturing advancements. The newly developed catheter will be integrated into EDEN2020’s ecosystem for precision neurosurgery. In addition, we plan to investigate new artificial intelligence (AI) applications, control schemes, and haptic teleoperation methods for needle steering. We acknowledge the potential benefits of combining machine learning and AI with needle-steering technology. By utilizing AI approaches (e.g., [47,48]), we aim to model steering behaviour with heterogeneous tissue structures and enhance the real-time control and adaptive steering capabilities for our catheter. This integration of AI holds great promise for improving the precision and effectiveness of neurosurgical interventions.

We have identified important limitations affecting the current prototype and proposed a comprehensive research strategy to address these challenges. Our objective is to conduct extensive investigations on material options, cutting angles, and preform moulding techniques, which have the potential to result in superior steering capabilities for our catheters. These advancements have the potential to improve the success rate of neurosurgical procedures and advance the field of minimally invasive surgery, especially for drug delivery.

The size reductions that have been achieved and current research projects show significant potential for improving the effectiveness of neurosurgical procedures. We hope to contribute to the ongoing innovation and advancement in the field of medical interventions by resolving the issues identified and pursuing the suggested future approaches, eventually applying our needle-steering technology to LiTT and drug delivery applications.

## Figures and Tables

**Table 1:** Mechanical feature test results for EM and TD catheters.

	Mean Flexural Stiffness (N/mm)	Mean Tensile Stress (MPa)	Mean Interlocking Breakout Force (N)
**2.5 mm** **EM**	0.023	13.10	5.47
**2.5 mm** **TD**	0.38	53.66	18.52
**1.3 mm** **TD**	0.031	51.45	10.94

**Table 2:** Single-segment insertion offset–curvature relationship.

	Offsets (mm)	Mean κ (1/mm)	Mean R (mm)	Mean β (degree)
**EM PBN**	5	0.0066	151.488	28.89
	10	0.0120	83.306	42.68
	15	0.0193	52.454	54.58
	20	0.0242	41.307	69.20
**TD PBN**	5	0.0026	385.516	8.93
	10	0.0052	192.432	19.24
	15	0.0073	136.410	28.12
	20	0.0092	109.113	34.30

**Table 3:** Two-forward-segment insertion offset–curvature relationship.

	Offsets (mm)	Mean κ (1/mm)	Mean R (mm)
**EM PBN**	5	0.0055	181.488
	10	0.0080	125.036
	15	0.0102	98.034
	20	0.0121	82.644
**TD PBN**	5	0.0025	400.056
	10	0.0037	270.270
	15	0.0053	188.679
	20	0.0069	144.921

## Data Availability

The data presented in this study are available on request from the corresponding author. The data are not publicly available due to privacy.

## Abbreviations

The following abbreviations are used in this manuscript:

MIS	minimally invasive surgery
CED	convection-enhanced delivery
TD	thermally drawn
EM	extrusion-manufactured
PBN	programmable bevel-tip needle
LiTT	laser interstitial thermal therapy
PC	poly carbonate
